# The SUMO guards for SNAIL

**DOI:** 10.18632/oncotarget.22432

**Published:** 2017-11-15

**Authors:** Xin Ye, Robert A. Weinberg

**Affiliations:** Department of Discovery Oncology, Genentech Inc., South San Francisco, California 94080, USA; Whitehead Institute for Biomedical Research, Cambridge, Massachusetts 02142, USA; Ludwig/MIT Center for Molecular Oncology, Cambridge, Massachusetts 02142, USA; Department of Biology, Massachusetts Institute of Technology, Cambridge, Massachusetts 02142, USA

**Keywords:** sumoylation, Snail, post translational modification, EMT, TGF-beta

The cell-biological program termed epithelial-mesenchymal transition (EMT) plays a key role in adenocarcinoma progression, invasion, and metastasis. An EMT operating within carcinoma cells can be activated by a variety of paracrine signals arising in the tumor microenvironment. These EMT-inducing signals trigger profound transcription changes through activation of EMT-inducing transcription factors (EMT-TFs), leading to repression of genes associated with the epithelial differentiation program and activation of those associated with the mesenchymal program.

The cytokine transforming growth factor-β (TGF-β) is one of the most potent and well-characterized EMT inducers. Binding of TGF-β to its cognate cell-surface receptors leads to phosphorylation and activation of the Smad transcription factors, which then translocate to the nucleus to activate TGF-β target genes including the Snail EMT-TF; the latter then proceeds to orchestrate many of the cell-biological changes associated with the EMT program. In our own hands, Snail, also known as Snai1, is involved in the malignant progression of mouse mammary carcinoma cells in a transgenic model of human mammary adenocarcinoma pathogenesis [1]. Thus, its ongoing functions appear to be critical to the invasiveness of high-grade carcinoma cells and their ability to seed new tumors, i.e., to function as cancer stem cells [1, 2].

Snail is unique among the cohort of EMT-TFs because it is strongly regulated at the post-translational level. While the activity of other EMT-TFs, such as Zeb1 and Twist, can be gauged by the levels of their respective mRNAs, Snail differs strongly, in that it may be degraded rapidly after its initial synthesis [3]. Like some other growth regulatory proteins such as β-catenin, Myc, and cyclin D1, the degradation of Snail is often initiated by phosphorylation by GSK-3β kinase, followed by poly-ubiquitination and destruction by the proteasome [3]. This phosphorylation by GSK-3β may be the main factor to ensure the relatively short half-life of Snail protein, its generally low steady-state levels, and its ability to increase rapidly in response to specific cell-physiologic stresses. In addition, Snail has been reported to undergo CREB-binding protein (CBP)-mediated acetylation in a head-and-neck cancer cell line [4]. In this latter case, the acetylation on lysine residues 146 and 187 of Snail stabilizes the protein and promotes the transcription-activating functions of Snail operating on some of its target genes.

The TGF-β-Snail axis is regulated by a variety of post-translational mechanisms. In particular, polyubiquitination can both suppress EMT by destabilizing Snail and, at the same time, enhance EMT through auto-ubiquitination of the E3 ubiquitin ligase Tumor Necrosis Factor Receptor-Associated Factor 6 (TRAF6) [5]. TRAF6, for its part, can promote proteolytic processing of the Type 1 TGF-β receptor (TβR1), liberating the TβR1-intracellular domain (TβR1-ICD), which can function as a Snail transcription activator when translocated into the nucleus [5].

In this issue of Oncotarget, Gudey et al. describe sumoylation as a new mechanism employed by TGF-β to activate Snail. Sumoylation represents a post-translational modification mechanism that is analogous to ubiquitination, involving the covalent conjugation of copies of a small ubiquitin-like modifier (SUMO) protein to target proteins. Similar to ubiquitination, Sumoylation is catalyzed by an enzymatic cascade involving three enzymatic steps catalyzed by the actions of an E1, E2, and E3 sumo ligase. Sumoylation has been shown to regulate nucleo-cytoplasmic transport, protein stability, chromosome organization, DNA repair, cell cycle progression, and other cellular processes [6]. Using the PC-3U prostate carcinoma cell line as a model system, Gudey et al show that TGF-β induces sumoylation of Snail at its K234 residue, which is critical for the EMT-activating function of Snail. Thus, the Snail K234R amino-acid replacement mutant loses its ability to undergo TGF-β-induced sumoylation in PC-3U cells, as well as its ability to promote migration and invasion as gauged by in vitro assays. Using in vitro sumoylation assays, they demonstrate that the K234 residue can be sumoylated by the SUMO isoform Sumo1 but not the isoform Sumo2. However, the ligases responsible for catalyzing these sumoylation reactions remain elusive. Lastly, the authors provide histology data showing co-expression of TGFβ1, Snail, and Sumo1 in malignant human prostate cancers. These data further strengthen the role of Snail as a key executor of the TGF-β-induced EMT program and establish a novel connection between EMT and sumoylation.

Intriguingly, the K234 residue of Snail is highly conserved across metazoan species and has been proposed to be in close contact with DNA. It is unclear whether sumoylation at this residue can contribute directly to the interaction between Snail and nearby chromatin-associated proteins. For example, the Snail protein has been found to form physical complexes with transcription repressors such as sin3A and LSD1, ostensibly enabling repressing of the epithelial genes [7]. Moreover, the functional implications of sumoylation await further investigation both in cultured cells and in vivo.
